# Opening Speech of Sir F. Thesiger

**Published:** 1852-04-01

**Authors:** 


### OPENING SPEECH OF SIR F. THESIGER.

Sir F. Thesiger then opened the case in support of the petitioners for the commission, and observed that the jury were assembled to inquire into the state of the mind of a lady of the advanced age of 73 years. In the discharge of his duty he felt it right to give them a simple narrative of the facts and circumstances of the case, abstaining from all comments until the whole evidence was before them. The unfortunate subject of their present inquisition was the only daughter of a Mr. Thomas Pritchard, a gentleman possessed of property in Wales. Under a marriage settlement of 1776, that property was settled upon himself for life, and afterwards in such a way that Mrs. Cumming, being his only child, would be entitled to an absolute interest in the estates. In 1808 Mrs. Cumming married a gentleman, who afterwards became a captain in the army, and in 1809 another settlement was made of the property—viz., to himself (the father) for life, and then to such uses as he should by deed or will appoint; Mrs. Cumming had by the same deed secured to her an annuity of 200*l.* during the lives of her father and mother, and the life of the survivor of them; and as, inasmuch as she was married, it was impossible that arrangement could be carried into effect without a fine being levied and a recovery suffered by her, she accordingly entered into a covenant that those instruments should be executed by her. The father of the lady died in 1811, having by his will, made in 1810, left all his property to trustees in trust to pay the rents and profits to his wife for life, and after her death to Mrs. Cumming; and upon her death the property was to be divided among her children. The jury would bear in mind the distinction between the settlement of the property under the original settlement of 1776 and the subsequent settlement of 1809—in the one case Mrs. Cumming would have the absolute interest in the Welsh property, while in the other case she would only have a life interest. There were two children of the marriage of Captain Cumming—both daughters. In 1833 the youngest daughter, Catherine Elizabeth, who was always the object of her mother's affection, married, with the approbation of her mother, a Mr. Ince, a surgeon. In 1837 the eldest daughter, Thomasine Catherine, married a person of the name of Hooper, a clerk in the Custom-house. This was without the approbation of Mrs. Cumming; but soon after the marriage she became reconciled to it, and received her daughter and son-in-law, and afterwards their children, with great affection; and ultimately appointed Mr. Hooper her agent and collector of her rents. This was the state of the lady's family in 1846. Should the jury consider Mrs. Cumming to be of unsound mind, it would be necessary for them to determine from what period that unsoundness had existed. It was not his intention to carry them back beyond the month of May, 1846. It might incidentally appear that prior to that time symptoms of derangement had taken place. She occasionally exhibited an alienation from her daughters; there was an intermission of all natural affection towards the members of her family, but that, he understood, was one of the commonest forms of insanity. In 1846, when Mrs. Cumming was living in Belgrave-place, Pimlico, with her husband, who was then of the advanced age of 88 or 89 years, so many instances of delusion manifested themselves that there existed no doubt whatever that her mind at that period was in an unsound state. However painful and disgusting it might be to enter into details, it would be necessary that he should characterize those delusions. Although her husband was of the advanced age he had described, and also in a state of great bodily infirmity, still Mrs. Cumming was possessed with a notion that he was continually having intercourse with her female servants and other persons who came to the house, and that she herself had on more than one occasion caught him in the fact. It was physically impossible that anything of this kind could have taken place, and, although she was reasoned with upon the subject, nothing could detach her mind from the persuasion of it. This led to violence of conduct towards her husband, which rendered it necessary that he should seek protection from the members of his family. Mrs. Cumming at the same time conceived a very strong aversion to both of her daughters. She believed they had conducted themselves improperly, and that they were in the habit of robbing her. This feeling of aversion extended itself to her grandchildren, although of tender age. She would often fall on her knees and call down the curse of God upon them. She believed that Mr. Ince had robbed her, and had destroyed one of the children of Mr. Hooper for the sake of his property. She thought a Mr. Dangerfield had also robbed her of money. She became very filthy in her habits. She attached herself to cats and birds, and, her husband being in a very infirm state of health, she frequently attempted to starve him. Sometimes she would use personal violence towards him, so that ultimately, in May, 1846, he was compelled to find shelter in the house of his son-in-law, Mr. Ince. It was quite evident from all this that this lady was not capable of taking care of herself or property, and that it was necessary to have recourse to law to protect her and others from violence. Accordingly, under the instructions of Captain Cumming, her husband, proceedings were taken to remove Mrs. Cumming to an asylum. She was conveyed to York-house, Battersea, where she was seen by two medical men—Mr. Wilmot and Mr. Johnson—who signed a certificate as to her state of mind. She was also visited by two of the Commissioners of Lunacy on May 19, 1846, who came to the conclusion that she was decidedly of unsound mind. Pending that commission, Captain Cumming died. The proceedings having originally been taken with his sanction, they were continued by the family after his death. He mentioned this circumstance, because he understood that the aversion felt by Mrs. Cumming towards her daughters would be attempted to be justified on the ground of these proceedings having been taken against her by them. The inquiry was originally conducted by a gentleman of the name of Farrer, but the jury would find that another gentleman was soon after introduced, whose name would become familiar to them in the course of this investigation, a Mr. Robert Haynes, at that time an articled clerk to a Mr. Robinson, but who was now that gentleman's partner. This firm was now conducting the present inquiry on behalf of Mrs. Cumming. An order was obtained for Mr. Robinson to appear in the former inquiry of 1846 for Mrs. Cumming, and under that order Mr. Robert Haynes attended the proceedings. But after three or four days, an arrangement was come to, which was agreed to by counsel on both sides. This agreement was a very important part of the case. The endorsement upon the briefs was this—

"Promoters to withdraw from further prosecuting this inquiry, stating that they had done so under an impression that it was desirable for all parties that an arrangement should be made; that an arrangement had been made that Mrs. Cumming should be immediately discharged from all restraint; arrangements to be made that three trustees should be appointed, in whom the property should be vested, one to be named by Mrs. Ince and Mrs. Hooper, another by Mrs. Cumming, and the third by the commissioner or the two trustees. A deed of settlement to be prepared, under which Mrs. Cumming should be entitled to rents and profits of estates for life. After her death one-third of the annual income to be held by trustees for the separate use of Mrs. Ince for life, after her death for the husband, if he survive her, and then to her children. Similar arrangements as to Mrs. Hooper. Mrs. Cumming to have power of appointment over remaining third: and if the power was not exercised, then such remaining third should be divided equally between Mrs. Ince and Mrs. Hooper."

The object of this arrangement undoubtedly was to reserve to Mrs. Cumming as much liberty as was consistent with her condition, and at the same time effectually to protect her property through the intervention of trustees; and if this arrangement had been faithfully carried out, there could be no doubt that this inquiry would have been wholly unnecessary. But from what subsequently occurred he was bound to state his conviction that it never was the intention that this arrangement should be honestly observed; but that the object was by any means to get rid of the commission, in order that this unfortunate lady might be left completely in the power of those who were determined, for their own advantage, to exercise an influence over her feeble mind, and which they had too successfully obtained by the course which they afterwards pursued. This arrangement having been entered into by instructions from Mr. Alfred Robinson, it would have been impossible for him to appear subsequently in any proceedings taken for the purpose of repudiating it, and accordingly the names of Carlon and Haynes were substituted for that of Mr. Robinson, and they immediately set to work to defeat the object for which the arrangement was entered into. The veil was rather a flimsy one; for Mr. Haynes was the brother of the Mr. Haynes who was the partner of Mr. Robinson, and he actively interested himself in all that took place subsequently to the arrangement. Mrs. Cumming was then removed from the sight of her family, and placed in the house of a Mr. Hutchinson. Being safely in the power of these parties, their first object was to obtain possession of the deeds which were deposited with Messrs. Saxon and Hooper, and without the possession of which no sale of her property could take place. It was soon discovered by those who had possession of Mrs. Cumming, that the fine and recovery which were necessary to give validity to the settlement of 1809 had never been levied or suffered; that settlement, therefore, was invalid, and the absolute interest which Mrs. Cumming derived under her father's settlement of 1776 still remained in force. To render that absolute interest available for their own purposes it was necessary to have possession of the deeds, and accordingly an action to recover them was brought. To that action there could be no defence, because the arrangement of 1846 was no answer to Mrs. Cumming's right. Immediately the deeds were obtained sales of her property took place to a great extent. That property was considered to be of the value certainly of above 20,000*l.*, and probably of 30,000*l.* It was learned from Mr. Robert Haynes that in 1851, by some means the miserable wreck of that property was 10,000*l.*, subject to a mortgage of 3000*l.*, which money had been received and used by some person or other. During the whole of this time the family were wholly unaware of the place where Mrs. Cumming was residing, and it was merely by accident that they at length discovered it. It seemed that, among other transactions which this lady was induced or compelled by Mr. Haynes to enter into, was to purchase two houses in Queen's-road, St. John's-wood. Those houses were let, one at 70*l.* and the other at 65*l.* a-year, there being a ground-rent of 15*l.* upon each house, and they had been valued together at 1000*l.*—certainly not more than 1200*l.*—and yet, according to Mr. Haynes, Mrs. Cumming was induced to purchase the equity of redemption for 1600*l.* But this was not all. Mrs. Cumming being in a precarious state of health, she might be removed by death; it therefore became necessary to provide against such a contingency. Mr. Haynes accordingly took care that a will should be made by which he secured to himself a legacy of 2000*l.*, and also a legacy of 2000*l.* to his wife; and he made himself sole residuary legatee. These matters had been discovered since the 1st of February, 1851. On the morning of that day, the police were going their rounds when they heard screams and cries of "Murder," proceeding from a house called Herbert-villa, Howley-road, St. John's-wood. In a little time Mrs. Cumming threw up the window and called the police, saying, "that her servant was going to murder her;" upon which they asked whether they could obtain admission, and she replied, "Oh, yes; my servant will let you in." They entered, and then the servant told them that her mistress was in a state of insanity. The police went up stairs, and found the bedroom door locked, and were obliged to use force to gain admittance. They talked a little with Mrs. Cumming, and, seeing no apprehension of danger, they left her in the care of her servants. Having thus become acquainted with the residence of Mrs. Cumming, witnesses would be able to describe the miserable and filthy condition in which that lady was found; and the strong feeling of alienation which existed in her mind towards her children. He was afraid there were persons studiously active in cherishing these delusions operating upon that subject in the mind of the lady. At that time Mrs. Cumming had employed a Mr. Thorne as her attorney, for the purpose of procuring the accounts and papers from Mr. Robert Haynes. The coachman employed by her was sent to Mr. Thorne, after the police had been to the house; but, instead of going to Mr. Thorne, the man went to Mr. Robert Haynes, and that person immediately took possession of the house and everything it contained. Mr. Thorne was not informed of Mrs. Cumming wishing to see him till the 3rd of February, and when he came he was denied access to that lady, and was told it was by Mr. Haynes's directions. On the 7th of February, he was told the furniture was being removed, and on going there he found that to be the fact. He saw Mr. Haynes, and remonstrated with him, upon which Haynes said it was done by his orders, and he would take the responsibility on himself. Part of the property was taken to Mr. Hutchinson's, and other parts elsewhere, and Mrs. Cumming was removed, but Mr. Haynes refused to tell where she had gone. However, Mr. Thorne discovered that she had been taken to Hutchinson's house, No. 104, Upper Stamford-street, Blackfriars. On the 11th of February he went there, but was told he could not be permitted to see her. In the course of the day he received a letter, signed by Mrs. Cumming and witnessed by Hutchinson, in these terms—

"Feb. 11, 104, Upper Stamford-street.

"Sir,—I request you will not call on me again, or trouble yourself with my affairs, as I have no wish to remove the papers from Mr. Haynes; and you will please to send in your account. "I am your obedient servant,

"Witness—S. Hutchinson." "Catherine Cumming.

On the following day the clerk of Messrs. Robinson and Haynes called upon Mr. Thorne with a letter, which letter was read to him, and of which a copy was taken. Curiously enough, that letter was dated on the 3rd of February. It was addressed to Mr. Robert Haynes, 17, Orchard street, Portman-square. It ran thus:—

"Sir,—It is my express wish and desire that you should continue the management of my affairs as my solicitor, and I request you will give no information respecting my affairs to Mr. Thorne, who, I am informed, has applied to you on the subject. You will also apply to him to furnish his account, in order that it may be paid. I take this opportunity of expressing my satisfaction at the manner in which you have managed my affairs, and to acknowledge that all your accounts with me have been examined and are correct. "I am, dear sir, yours truly,

"Catherine Cumming."

The family having learnt that Mrs. Cumming was residing at Stamford-street, her daughters called to see her, but they were refused admittance. It having been discovered where Mrs. Cumming was, it became dangerous to the parties interested for her to remain there; she was therefore clandestinely removed to the house of a Mr. Oldfield, in Edgeware-road, Mr. Oldfield being a clerk of Messrs. Robinson and Haynes. She was again discovered, and her daughter, Mrs. Ince, obtained an interview with her, when Mrs. Cumming received her with all the feelings of maternal affection. But Mr. Haynes very soon made his appearance, and desired Mrs. Ince to walk out, he refusing to allow her to remain without his presence; and she was compelled to leave. She called on the following day, and again saw her mother, in the presence of Mrs. Oldfield. Mrs. Cumming was relating to her daughter the utter neglect with which she had been treated, when Mrs. Oldfield began to stamp on the floor and scream violently; the servant came up, and it was impossible for Mrs. Ince to continue her visit. On the following day Mrs. Ince and Mrs. Hooper both went to Edgeware-road, but they were refused admittance; and Mrs. Cumming was again clandestinely removed, and her family lost sight of her for many months. They then applied to Mr. Turner, who was their solicitor, promoting this commission. That gentleman having ascertained that the Commissioners of Lunacy were aware of Mrs. Cumming's residence, he applied to them for information, and they told him that she had been removed to Worthing, under the feigned name of Cleveland, accompanied by a person of the name of Jones, under the assumed name of James. An officer of the police, who had been previously employed to discover Mrs. Cumming's residence, again found that she had been removed from Worthing to No. 5, Bloomsbury-place, Brighton. In consequence of a motion made before the Lord Chancellor, on the 27th of October last, Sir A. Morison and Dr. Monro were appointed to examine Mrs. Cumming. Sir A. Morison went down to Brighton, and, having power for that purpose, associated with him Dr. King, and they went to Bloomsbury-place. They were accompanied by the chief constable of Brighton, and, on their arrival at the house, who should appear but Mr. Robert Haynes, ready to receive them. He did all in his power to obstruct their access to the lady, and threatened he would bring an action against any person who ventured to use force to open the door of the bed-room in which Mrs. Cumming was stated to be. However, the door was opened, and Sir A. Morison and Dr. King saw Mrs. Cumming, in the presence of a Mrs. Watson. After a patient examination of that lady, during which all her delusions manifested themselves, those gentlemen came to the conclusion that she was a person of unsound mind, and incapable of taking care of herself and of her affairs. It was considered desirable that she should be placed under proper protection, and she was accordingly taken to an asylum at Effra-hall, Brixton, kept by Dr. Pettigrew. It had been asserted that violence had been used towards her on that occasion, but he could prove that no violence was used to remove her from Brighton. When she arrived at the station, Hutchinson and James endeavoured to drag her away from the nurse who had her in charge, and who was obliged to entrust her to the care of the police while she went to procure a railway carriage. But, on reaching Dr. Pettigrew's, it was found she had not suffered in the slightest degree. In the asylum she had been seen by several medical gentlemen, all of whom were of opinion she was of unsound mind; but after a little while a petition was presented to the Lord Chancellor, by Mr. Robert Haynes, to request she might be removed from the asylum, and allowed to reside in one of her own houses. Accordingly she was permitted by the Lord Chancellor to reside at the Gothic Villa, attended by Mrs. Moore, a lady selected by Mr. Robert Haynes, and who had great influence over her, and might possibly, by the moving of a finger, prevent her from speaking in the presence of medical gentlemen of those delusions which her mind laboured under. It was under all these circumstances that this inquiry was instituted, and, unless the whole case he had been instructed to state was a gross exaggeration, and in part a fabrication, he had not any doubt that this lady was not only of unsound mind in 1846, but that she had continued uninterruptedly so to the present moment. They would undoubtedly hear on the other side the evidence of gentlemen of science and character, who would express their opinion that she was not of unsound mind. He was anxious that the jury should carefully weigh testimony of that description, but they would have to exercise what he would venture to call a common-sense judgment, which sometimes was worth all the skill and science in the world in estimating the comparative value of the opinions which they would hear on one side and on the other. Having now stated the case to them, he would proceed to call his witnesses.

